# Iron, manganese, cadmium, chromium, zinc and arsenic groundwater contents of Agbor and Owa communities of Nigeria

**DOI:** 10.1186/s40064-015-0867-0

**Published:** 2015-03-01

**Authors:** Hector Henry Oyem, Ifeanyi Mirian Oyem, Amii Isaac Usese

**Affiliations:** Department of Integrated Science, College of Education, Agbor, , PMB 2090, Delta State Nigeria; School of Chemistry, Newcastle University, Newcastle, , NE1 7RU, , United Kingdom; Department of Microbiology, University of Benin, Benin City, Edo State Nigeria; Department of Marine Sciences, Faculty of Science, University of Lagos, Akoka-Yaba, Lagos, Nigeria

**Keywords:** Ground water, Boji-Boji area, Heavy metals, Chronic daily intake, Hazard Index, Guideline value

## Abstract

Iron, manganese, zinc, cadmium, and chromium heavy metals and arsenic contents of groundwater in area and surrounding environment, of Ika land in the Delta state, Nigeria was studied. Groundwater without any treatment is the predominant source of drinking water by inhabitants of these communities. However, the quality of this water source is not immediately known, raising questions of its safety.

Results of a regional composite of groundwater obtained, shows high iron (27%) and zinc (36%) contents in Boji-Boji Agbor area, manganese (31%) was highest in Boji-Boji Owa. Alihame recorded the lowest value of zinc (8%), while manganese was lowest in Agbor Obi area (12%). Arsenic, cadmium, and chromium were below detection limit in all the sample sites. Correlational matrix analysis revealed no significant relationships between metal types studied. Analyses of chronic daily dose intake (CDI), and hazard index were all very low. A hazard index of 0.01 was obtained. One-way ANOVA show significant statistical difference in the mean concentrations of the heavy metals for the different sample sites, which indicate that different sites contribute differently to the mean concentrations of the groundwater in the study area.

Four conclusions are drawn from this study. Indications are that the heavy metals present in the Nigerian aquifer are very much below the maximum concentration levels and guideline values of national and WHO standards. Secondly, there is a heavier load of these metals in the city centre than in the suburbs; with Boji-Boji area Agbor/Owa urban areas accounting for 27 and 20 percent of load respectively. Thirdly, the below detection limit results for some of the metal ions and the very low concentrations of those detected are pointers to the absence of industrial activities and mining. Finally, the groundwater in the study area is considered to be generally safe with respect to the contaminants studied and results posted for the composite samples. Inhabitants are therefore under no illusion of immediate or remote health challenges with regards to the heavy metals analyzed. More individual sampling, however, is recommended.

## Background

Water is a precious and most commonly used resource (Ramesh and Elango 2014). It is one of the most abundant chemical substances on earth, and covers two thirds of the earth’s surface (Ramesh and Elango 2014). According to Bresline (2007) and National Academy of Science NAS (2009), over one billion people lack access to clean safe water. The available freshwater to man is hardly 0.3 - 0.5% of the total water available on the earth and its judicious use is imperative (Ganesh and Hedge 1995). Many people in the world especially majority of which live in rural areas among the poorest and most vulnerable do not have access to safe clean drinking water (MacDonald and Calow 2009). In a recent survey by Majuru et al. (2011) an estimated 65 million Nigerians had no access to safe water. Provision of clean, reliable and portable water in rural areas and urban slums remains a challenge to governments throughout the world especially considering the fact that larger percentage of the population live in the urban areas (Ahaneku and Adeoye 2014). Without clean water, people’s health and livelihoods can be severely affected (MacDonald and Calow 2009).

The burden of providing water close to consumers is met by boreholes that tap groundwater. These boreholes are typically deep (more than 100 m), narrow, mechanically-drilled wells fitted with electric pumps to tap groundwater reservoirs.

Groundwater is a vital hidden natural resource (Tularam and Krishna 2009; Lashkaripour and Ghafoori 2011). Groundwater can be found in most environments and generally requires no prior treatment and can be found close to the points of demand often at low cost (MacDonald and Calow 2009). Traditionally regarded as being of good natural quality mostly from its geological environment, this does not mean that natural groundwater is always of good quality (MacDonald and Calow 2009). Water in its natural state may not be pure since it is a universal solvent with the ability to dissolve numerous chemicals and thus carry a lot of impurities that can be injurious to health if it exceeds tolerable limits World Health Organization WHO 1984. Consequently the, groundwater resource itself is not invulnerable given the ability to pump out large quantities of water and the advent of particularly persistent contaminants (MacDonald and Calow 2009). The natural quality can vary from one rock type to another and also within aquifers along its flow paths. There is also the possibility for chemical reactions between the water and rock material through which it flows depending on the flow path (Lashkaripour and Ghafoori 2011), especially since groundwater movement is slow (MacDonald and Calow 2009). Groundwater quality can deteriorate through contamination of the local groundwater, or direct contamination of the water supply itself (MacDonald and Calow 2009). Contaminants can migrate vertically to the aquifer and then to the borehole, or more dangerously, horizontally through permeable soils to poorly constructed supplies (MacDonald and Calow 2009).

Consistent from findings reported in literature is that groundwater is polluted from physical processes and anthropogenic activities (Idoko 2010). Among the chemical contaminants of groundwater are some heavy metals. Drinking groundwater and surface water contaminated by heavy metal ions is detrimental to health (Ohwoghere 2012). Metallic contaminants are serious concerns in many water bodies around the world United Nations Environmental Programme Global Environmental Monitoring System, (United Nations Environmental Programme Global Environmental Monitoring System and UNEP 2007). Many potentially deadly diseases associated with groundwater consumption have been traced to heavy metal contaminants. Interestingly though, some of these same metals are required by the body in trace quantities. However, large doses especially over the course of time are inimical to health.

Water is essential for life but it does transmit diseases in countries in all continents – from the poorest to the wealthiest (World Health Organization WHO 2010). Millions of people are exposed to unsafe levels of chemical contaminants in their drinking water (World Health Organization WHO 2010). Monitoring metals in surface or groundwater supplies provides background information needed to determine the suitability of water resources for human consumption (United Nations Environmental Programme Global Environmental Monitoring System and UNEP 2007).

In Boji-Boji area of Agbor/Owa communities in Delta State Nigeria, groundwater is the dominant source of water for the people. This resource is flagrantly consumed without recourse to its quality. We are not immediately aware of any previous study on the groundwater quality of this area. It is imperative to understand the groundwater resources to ensure it is fit to drink and to protect the water supply from contamination (MacDonald and Calow 2009). Urgent need for water prioritizes borehole development over scientific study of groundwater quality. The continued consumption of untreated and possibly contaminated groundwater should be expected to pose short or long term (or even both) health implications to the people.

The drinking water guidelines applied in this study are the Nigerian Industrial Standard (NIS), World Health Organization (WHO), United States Environmental Protection Agency (USEPA), and the European Union (EU) as presented in Table 1. However, emphasis is on the Nigerian and WHO guideline values presented in Table 1.

**Table 1:** **Summary of Nigerian and some international guideline values for drinking water samples**

**Parameter** **(element/** **substance)**	**Nigerian guideline value** **(mg/** **L)**	**WHO guideline value** **(mg/** **L)**	**USEPA guideline value** **(mg/** **L)**	**EU guideline value** **(mg/** **L)**
**Arsenic** **(As)**	0.01	0.01	0.01	0.01
**Barium** **(Ba)**	0.7	0.7	2.0	NA
**Cadmium** **(Cd)**	0.003	0.003	0.005	0.005
**Chlorine** **(Cl** ^**−**^ **)**	250	250	250	400
**Chromium** **(Cr** ^**6+**^ **)**	0.05	0.05	0.1	0.05
**Chemical Oxygen Demand**	NA	NA	NA	NA
**Conductivity** **(μS/** **cm)**	1000	NA	NA	NA
**Cyanide** **(CN** ^**−**^ **)**	0.01	0.07	0.2	0.05
**Hardness** **(as CaCO**_**3**_ **)**	150	180	NA	NA
**Iron** **(Fe** ^**+2**^ **)**	0.3	0.3	0.3	0.2
**Manganese** **(Mn** ^**2+**^ **)**	0.2	0.4	0.05	0.001
**Mercury** **(Hg)**	0.001	0.006	0.002	0.001
**Nitrate**	50	50	10	50
**Ph**	6.5-8.5	6.5-8.0	6.5-8.5	5.5-9.5
**Phosphate** **(PO**_**4**_^**3-**^ **)**	NA	NA	NA	NA
**Sodium** **(Na)**	200	200	NA	150
**Sulphate** **(SO**_**4**_^**2−**^ **)**	100	250	250	250
**Total Dissolved Solids**	500	NA	NA	NA
**Zinc** **(Zn)**	3.0	3.0	5.0	NA

*NA* No guideline available.

Human and natural factors have been noted as making it difficult for these guideline values to be maintained generally (United Nations Department of Economics and Social Affairs and UNDESA 2001).

In this exercise, we study some heavy metal characteristics in the groundwater from boreholes in Boji-Boji Agbor/Owa town and its immediate suburbs of Alihame and Owa Alero communities with the intention of evaluating its quality.

### Study area

**Figure 1:**
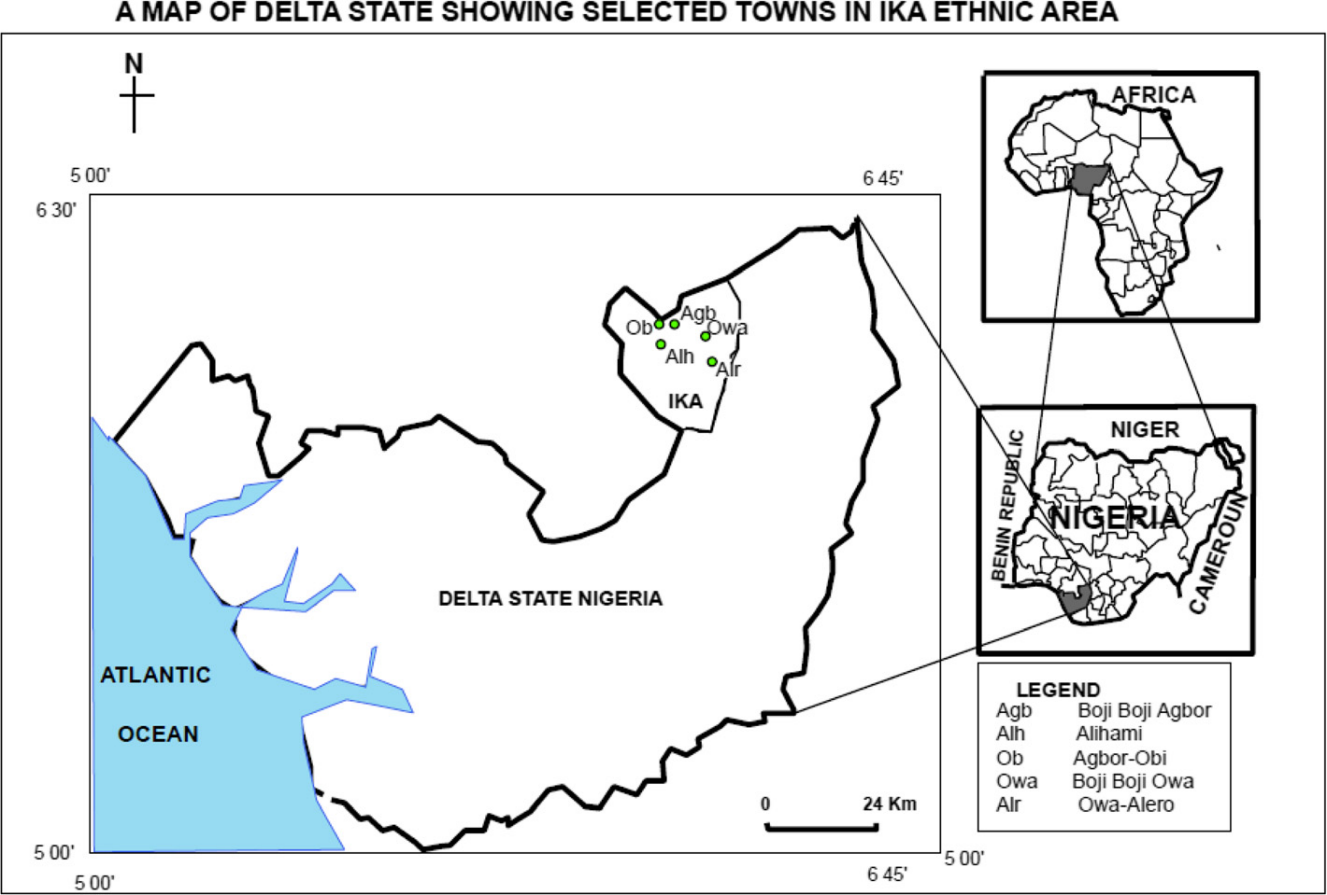
**Map of Delta State Nigeria showing sample sites.** Source: Department of Land Information Systems, Ministry of Lands Surveys and Urban Development, Asaba Delta State.

The study area (Agbor/Owa town commonly referred to as *Boji*-*Boji*) found within longitudes 6°- 6° 30’ E and latitudes 6°- 6° 45’ N, was mapped out in to five (5) sub-areas of Agbor Obi, Boji-Boji Agbor, Boji-Boji Owa, Alihame, and Owa Alero (see Figure 1).

The latter two areas (Alihame and Owa Alero) being classified as suburban towns for the purpose of this exercise on the basis of socio-economic stratification and population density. The geology of the area is mainly of the recent tertiary sedimentary sandstone with fine to coarse reddish (iron, haematite) sediments in texture giving it a porous nature, with the Bini formation as a typical example. This indicates a lee way for easy passage of leachate (like iron) in to the groundwater in the underlying aquifer.

The climate of the study area exhibits the characteristics of a sub-equatorial climate with an annual mean air temperature of 27.0 °C (Odjugo 2008). The rainfall pattern is that of double peaks or maximal with mean annual rainfall of 2,255 mm, while the mean relative humidity is 81%, sunshine is 5.6 hr/day and the soil type is red-yellow ferralsols (Avwunudiogba 2000).

### Experimental

A total of 50 borehole water sources was sampled from these areas, with an average of about 12–15 per area for Agbor Obi, Boji-Boji Agbor and Owa areas, and five (5) for Owa Alero and Alihame communities on the basis of fewer wells per unit area. Samples were collected in clean new 300 ml sterile bottles with corks (Burubai et al. 2007). Borehole water sample sites were randomly spaced and samples collected in sterile bottles filled to the brim, then preserved by cooling in dark ice bag (Tomar, 1999) before taken to the laboratory for analysis immediately after sampling without filtration and acidification (EPA 2000).

Analyses of samples were done in the laboratories of the College of Education Agbor and at a private laboratory. At the laboratory, the samples were carefully transferred in to a clean 4 L container and a composite sample (Patil 2012) was thus formed per sample sub-area. An average of 12–15 individual borehole water samples were combined in to a composite per sub-area in the three main sub-areas. One sub-area at a time was sampled. These composites were analyzed each in triplicates along with a blank (distilled deionized water, DDW) using Solar Unicam Atomic Absorption Spectrophotometer (United Nations International Children’s Emergency Fund UNICEF 2008) model 969 series, with a detection limit of 0.0001 mg/L and limit of quantification of 0.0003 mg/L respectively. Methods were consistent with APHA (2005) and (United Nations International Children’s Emergency Fund UNICEF 2008).

All samples were collected in the mornings in the month of August, 2013 during the wet season, with rainfall at its peak in the months of July/August in southern Nigeria (UNOCHA 2013).

Typical water parameters like pH, total dissolved solids, turbidity, conductivity, and cyanide and other anions and cations together with some microbiological parameters were all studied, some of these are already published (Oyem *et a*l., 2014).

## Results and discussion

The results of selected heavy metals in groundwater of the study area are displayed in Table 2. Generally, the studied metal ions concentration were below WHO guideline values.

**Table 2:** **Presents the concentrations of selected heavy metals in the ground water of the study area**

**Sample Area**	**Fe** **(mg/** **L)**	**Mn** **(mg/** **L)**	**Zn** **(mg/** **L)**	**Cr** **(mg/** **L)**	**As** **(mg/** **L)**	**Cd** **(mg/** **L)**
**Agbor Obi**	0.090	0.007	0.019	BDL	BDL	BDL
***Boji***-***Boji*** **Agbor**	0.130	0.011	0.028	BDL	BDL	BDL
***Boji***-***Boji*** **Owa**	0.090	0.018	0.010	BDL	BDL	BDL
**Owa Alero**	0.070	0.009	0.015	BDL	BDL	BDL
**Alihame**	0.110	0.013	0.006	BDL	BDL	BDL
***Mean***	***0.100***	***0.010***	***0.020***	BDL	BDL	BDL

*BDL* Below detection limit (<0.0001 mg/L).

### Iron

Iron is a secondary priority chemical contaminant (United Nations International Children’s Emergency Fund UNICEF 2008). The concentration of iron from the sampled areas show very distinctly that the largest concentration of iron (0.13 mg/L) representing 26.5% (Table 3) was obtained at Boji-Boji Agbor sample sub-area, this is typical of the area bearing in mind that the commercial activities of automobile mechanics and iron/metal works artisans, and carcasses of abandoned and unserviceable cars are strewn about.

**Table 3:** **Comparative analysis of iron contents of individual sample sub**-**areas relative to WHO maximum contaminant level**

	**Agbor Obi**	***Boji***-***Boji*** **Agbor**	***Boji***-***Boji*** **Owa**	**Owa Alero**	**Alihame**
Fe detected (%)	18.4	26.5	18.4	14.3	22.5
Minimum conc. Detected (mg/L)	0.08	0.12	0.08	0.06	0.10
Maximum conc. Detected (mg/L)	0.10	0.14	0.10	0.08	0.12
WHO Maximum Contaminant Level (MCL) (mg/L)	0.30	0.30	0.30	0.30	0.30
Number above MCL	0	0	0	0	0

Percentage heavy metal detected is an expression of the concentration of these metals in a sample sub-area relative to the total concentration of the same metal in the entire study area in percentage term. Boji Boji Agbor is highest in concentration of Fe (26.50%). Alihame was next with an iron concentration of 0.11 mg/L (22.5%) which is rather surprising giving its rural setting. However, this may not be unconnected with the hydrogeological nature of the area. Owa Alero (0.07 mg/L) was the least Fe (14.3%). In all, the study area was observed to have a mean iron concentration of 0.10 mg/L. Iron is frequently found in groundwater due to large deposits in the earth’s surface; however, a limit of 0 – 0.3 mg/L is acceptable (Edet *et al*. 2011). The levels of Fe in groundwater can be increased by dissolution of ferrous borehole and hand pump components (Lenntech 2009). Results of Fe and Mn reported in this study generally agree with those reported by Edet *et al*. (2011).

The use of groundwater for drinking is in many cases limited by the presence of dissolved iron, and to a lesser extent, manganese. These give the water an unpleasant metallic taste, and stain food, sanitary wares and laundry (United Nations International Children’s Emergency Fund UNICEF 2008). Therefore the results obtained for the iron content of the groundwater in this study are rather encouraging, and suggests portable water quality with reference to the metals under study; especially as values recorded are all far below the Nigerian (Nigerian Industrial Standard 2007), WHO, United States Environmental Protection Agency (USEPA) and European Union (EU) guideline values.

### Manganese

Manganese like iron is a secondary priority chemical contaminant. The values derived for manganese are expressed in Table 2 as well. Boji-Boji Owa recorded the highest manganese value of 0.018 mg/L representing 31.0% (Table 4).

**Table 4:** **Comparative analysis of manganese contents of individual sample sites in the study area**

	**Agbor Obi**	***Boji***-***Boji*** **Agbor**	***Boji***-***Boji*** **Owa**	**Owa Alero**	**Alihame**
Mn detected (%)	12.1	19.0	31.0	15.5	22.4
Minimum conc. Detected (mg/L)	0.006	0.008	0.016	0.008	0.012
Maximum conc. Detected (mg/L)	0.008	0.015	0.020	0.010	0.014
WHO Maximum Contaminant Level (MCL) (mg/L)	0.400	0.400	0.400	0.400	0.400
Number above MCL	0	0	0	0	0

This again is expected in view of the economic density of automobile and allied metal activities in the Boji-Boji area. 0.013 mg/L (22.4%) was the second highest value obtained for Alihame. Again, Alihame showing high metal ion content with reference to Fe and Mn indicating a natural hydrogeologic factor rather than anthropogenic. Boji-Boji Agbor posted a significant value when compared with those of Owa Alero and Agbor Obi (with the least value of all sampled areas). However the entire study area reported an average manganese value of 0.010 mg/L. This again is far lower than the guideline value recommended by Nigerian, WHO, EU, and USEPA, indicating yet again palatability quality wise, since it is one of the secondary priority chemical contaminants, and also responsible in this case also for water being rejected for aesthetic purposes (United Nations International Children’s Emergency Fund UNICEF 2008).

### Zinc

Zinc was observed in all the sub-areas of the study as can be seen in Table 5. However, an average concentration of 0.02 mg/L was determined for the area under study. 0.028 mg/L obtained in Boji Boji Agbor was the highest value noted in the entire sampled areas, representing 36.0% (Table 5). All others were below 0.020 mg/L (0.19, 0.015, 0.010, and 0.006 mg/L). The result obtained in Boji Boji Agbor could be attributed to the fact that zinc being a constituent of roofing sheets, has been washed down by rainfall into the soil before ending up in the underground water by leaching over decades of time in these semi-urban areas. Alihame recorded near zero concentration. Nonetheless, it is speculated that the zinc content of this study area could be attributed to natural causes rather than human activities. Zinc imparts an undesirable astringent taste to water at concentrations exceeding 3 mg/L (as ZnSO_4_). However, drinking water seldom contains zinc above 0.1 mg/L. Pipe line water supply with galvanized plumbing material records higher levels of zinc (World Health Organization WHO 2010). More so, an average zinc value of 0.02 mg/L observed for the study area is acceptable, since both the Nigerian and WHO guideline is set at 3.0 mg/L for zinc in drinking water.

**Table 5:** **Comparative analysis of zinc contents of the study area**

	**Agbor Obi**	***Boji***-***Boji*** **Agbor**	***Boji***-***Boji*** **Owa**	**Owa Alero**	**Alihame**
Zn detected (%)	24.4	36.0	12.8	19.2	7.7
Minimum conc. Detected (mg/L)	0.017	0.027	0.009	0.013	0.005
Maximum conc. Detected (mg/L)	0.019	0.029	0.012	0.017	0.008
WHO Maximum Contaminant Level (MCL) (mg/L)	3.000	3.000	3.000	3.000	3.000
Number above MCL	0	0	0	0	0

### Arsenic, cadmium and chromium

Arsenic, cadmium, and chromium were all *below detection limit* in the groundwater of the study area. This implies that the groundwater in the study had no traces of the metal ions being mentioned, or that their concentrations were just too low to be detected by the instrument of analysis. This is good news, since cadmium and chromium might be considered a threat, and certainly arsenic in drinking water is a global threat to health (United Nations International Children’s Emergency Fund UNICEF 2008; World Health Organization WHO 2010). None of these metals were detected in water from all five sampled areas collected for analysis.

### Chromium

Chromium occurs in several forms in the environment, most of which are Cr^3+^ and Cr^6+^ with varying health importance; but drinking water standards are typically made for total chromium (United Nations International Children’s Emergency Fund UNICEF 2008). Concentrations of chromium in natural water is usually low, however, elevated concentrations result from mining and industrial processes (Momodu and Anyakora 2010). Chromium (VI) has severe health impact (United Nations International Children’s Emergency Fund UNICEF 2008) when inhaled. However, there’s no evidence of its carcinogenicity when taken orally (Flegal and Last 2001). Hence its health impact is still a subject of controversy. A guideline value of 0.05 mg/L is given to chromium by the WHO.

The non-detect result for chromium in this study is an indication and likely confirmation of the absence of mining and other forms of industrial activities.

### Cadmium

Cadmium metal is used in steel, plastic, and battery industries (World Health Organization WHO 2010). Its presence in drinking water could be as a result of impurities from galvanized zinc pipes and solder, together with other metal fittings. Cadmium accumulates in the kidney being its target organ of toxicity (World Health Organization WHO 2010). Cadmium is potentially carcinogenic in humans; it is the cause of itai itai disease observed in Japan due to excess intake (Lauwerys 1979). It has long biological half-life of 10–35 years in humans (Orisakwe, et al. 2006) leading to chronic effects as a result of accumulation in the liver and renal cortex (Hammer and Hammer 2004). Although there is no evidence of its carcinogenicity, the WHO has a guideline value for Cadmium of 0.003 mg/L.

### Arsenic

Arsenic in water is mostly present as arsenate (5+), but in anaerobic conditions, it is likely present as arsenite (3+) (World Health Organization WHO 2010). Arsenic in drinking water is a global threat to health (United Nations International Children’s Emergency Fund UNICEF 2008; World Health Organization WHO 2010). It is considered by some researchers to have more serious health repercussions than any other environmental contaminant (Smith and Steinmaus 2007). Signs of chronic exposure to arsenic, including dermal lesions such as hyper-and hypo-pigmentation, peripheral neuropathy, skin cancer, bladder and lung cancers and peripheral vascular disease, have been observed in populations ingesting arsenic-contaminated drinking water (World Health Organization WHO 2010); with both As^3+^ and As^5+^ rapidly and extensively absorbed from the gastrointestinal tract. Although arsenic contaminations occur in surface water, it is more common in ground water (United Nations International Children’s Emergency Fund UNICEF 2008), where arsenic remain tightly bound to sediments under geochemical conditions, and dissolved levels remain very low until released by the parent rock. There is no effective treatment for chronic arsenic poisoning, except for switching to arsenic-free drinking water source (United Nations International Children’s Emergency Fund UNICEF 2008). A guideline value of 0.01 mg/L is given to it by the (World Health Organization WHO 2010).

Table 6, gives a picture of the total detected heavy metal content of the individual study sub-areas in percentage. Noticeably, that the highest concentration (~27 per cent) of heavy metals in the groundwater is concentrated in the Boji-Boji Agbor area. Both Boji-Boji Agbor and Owa areas being the centre of town and hub of commercial and sociological activities has a high combined heavy metals capacity of close to 50 percent of the total study area. However, a striking report of note is the total heavy metals content determined for Alihame. Although, considered a suburban area, it is nonetheless a budding commercial hub and residential area, hosting students of the College of Education, Agbor, and State School of Nursing, Agbor, as well as staff of these institutions, civil servants and indigenous settlers.

**Table 6:** **Iron**, **manganese**, **and zinc metals combined distribution in groundwater of the individual sample sites in the study area**

**Sample site**	**Fe** **(mg/** **L)**	**Mn** **(mg/** **L)**	**Zn** **(mg/** **L)**	**Total** **(mg/** **L)**	**Percentage (%)**
**Agbor Obi**	0.090	0.007	0.019	0.116	*18.53*
**Boji**-**Boji Agbor**	0.130	0.011	0.028	0.169	*26.99*
**Boji Boji Owa**	0.090	0.018	0.010	0.118	*18.85*
**Owa Alero**	0.070	0.009	0.015	0.094	*15.02*
**Alihame**	0.110	0.013	0.006	0.129	*20.60*
				*Total* = *0.626 mg*/*L*	

Analysis of variance (ANOVA) is designed for simultaneous testing of equality of three or more populations of independent groups to determine if each group mean is identical assuming each has a normal distribution around its mean (Helsel and Hirsch 2002). Therefore in this study, to ascertain if data from the sampled sub-areas are identical or significantly different, we employed the use of a single factor ANOVA tool (Helsel and Hirsch 2002; Muhammad et al. 2011).

Table 7, presents a one-way analysis of variance (ANOVA) statistical comparison of selected heavy metal contaminants from the different sample sub-areas. Results indicate that the groundwater from individual sample sub-areas are individually different from each other and are thus not the same. This obviously shows that different sites contribute differently to the mean heavy metal concentrations of the water in the study areas’ aquifer. Variations in this regard probably arise from changes along the groundwater flow path, and slight variations in net effect of the pH dependent processes of minerals dissolution and precipitations (hydrogeology).

**Table 7:** **One**-**way ANOVA comparison of detected heavy metal contaminants in the study area**

**Metals**		**Sum of squares**	**df** ^**a**^	**Mean Square**	**F** ^**b**^	**P**-**value**
**Fe**	Between group	0.0063	2	0.0032	38.4	0.00001
	Within group	0.0010	12	0.000083		
	Groups total	0.0073	14			
**Mn**	Between group	0.000252	2	0.000126	16.0	0.0004
	Within group	0.000100	12	0.000008		
	Groups total	0.000352	14			
**Zn**	Between group	0.001158	2	0.000599	217.8	0.0
	Within group	0.000033	12	0.00000275		
	Groups total	0.001191	14			

P < 0.05 ^a^Degree of freedom ^b^Factor.

### Chronic daily intake

Health risks associated with ingestion of heavy metals in the groundwater of this study area are assessed using the chronic daily intake and hazard index parameters. The CDI through water ingestion was calculated according to the modified equation from USEPA (1992) and Chrostowski (1994):

1 $$ CDI=C\kern0.5em x\kern0.5em DI/BW $$

Where, C, DI, and BW represent the concentration of heavy metals in water (μg/L), average daily intake rate (2 L/day) and body weight (72 kg), respectively (USEPA 2005).

Results suggest that groundwater in the study area contains some dissolved heavy metals. CDI values obtained ranged from 0.02 to 0.13 for Fe, 0.0002 to 0.0005 for Mn, and 0.0002 to 0.0008 for Zn. Therefore, the order of heavy metal mean toxicity for the groundwater of the study area is Fe > Mn > Zn respectively; with Cd, Cr, and As none detected. These CDI values, however, give an indication of possible toxicity of these heavy metals found in the aquifer of the area. Since the CDI is below the reference dose, (RfD) values (USEPA 2005), it is assumed that the risk of consuming water with this heavy metals concentration is negligible for all members of an exposed population.

### Hazard quotient

The hazard quotient (HQ) for non-carcinogenic risk can be calculated from equation:

2 $$ HQ=CDI/RfD $$

(Gerba 2001, and USEPA 1999)

Table 8 also gives a summary of the hazard index on the health of the people of this area through regular consumption of groundwater. The mean hazard index values for the studied heavy metals ranged from 0.001 to 0.006 for Fe, 0.001 to 0.004 for Mn, and 0.001 to 0.003 for Zn respectively, the order of toxicity being Fe > Mn > Zn. Meanwhile, the hazard index calculated from the sum of the mean hazard quotients of the contaminants (eqn 3):

**Table 8:** **Chronic daily intake and Hazard quotient indices with Reference dose for studied heavy metals**

**Heavy metals**	**Statistics**	**CDI mg/** **kg**-**day**	**HQ**	**RfD mg/** **kg**-**day** ^**a**^
Fe	Min 0.07 Max 0.13 Mean 0.10 SD 0.023	0.002 0.004 0.003 0.0006	0.003 0.006 0.004 0.0009	0.7
Mn	Min 0.007 Max 0.018 Mean 0.010 SD 0.005	0.0002 0.0005 0.0003 0.0004	0.014 0.004 0.002 0.003	0.14
Zn	Min 0.006 Max 0.028 Mean 0.020 SD 0.009	0.0002 0.0008 0.0006 0.0003	0.0007 0.003 0. 002 0.001	0.3
Cd	BDL	BDL	BDL	0.0005
Cr	BDL	BDL	BDL	0.015
As	BDL	BDL	BDL	0.0003

*BDL* Below detection limit.

^a^RfD (USEPA 2005).

3 $$ \mathrm{Hazard}\;\mathrm{Inde}{\mathrm{x}}_{\mathrm{mean}}=\mathrm{H}{\mathrm{Q}}_{\mathrm{Fe}}\kern0.5em +\kern0.5em \mathrm{H}{\mathrm{Q}}_{\mathrm{Mn}}\kern0.5em +\kern0.5em \mathrm{H}{\mathrm{Q}}_{\mathrm{Zn}} $$

(United States Environmental Protection Agency and US 2006)

$$ =0.004+0.002+0.002 $$

$$ \mathrm{Hazard}\kern0.5em \mathrm{Inde}{\mathrm{x}}_{\mathrm{mean}}=\mathbf{0.008}\kern0.5em \left(\mathbf{0.01}\kern0.5em \mathbf{approx}.\right) $$

Therefore, since the hazard index is less than 1.0 (Khan *et al*. 2008, Krishna and Mohan 2013), the water is confirmed as being safe with reference to the studied parameters and results reported.

### Correlational analysis

Result of the correlational matrix as displayed in Table 9 did not reveal any strong or significant inter-metal relationships in the groundwater under study; hence it does not provide enough information on metal sources and pathways (Manta et al. 2002).

**Table 9:** **Correlation matrix of selected heavy metals in groundwater of the study area**

**Correlations**			
	**Fe**	**Mn**	**Zn**
**Fe**	1	0.146	0.407
**Mn**	0.146	1	−0.493
**Zn**	0.407	−0.493	1

## Conclusion

This study looked in to the groundwater aquifer quality at the centre of Ika land with specific reference to the Boji-Boji Agbor/Owa metropolis and its immediate suburbs Alihame and Owa Alero.

Of the heavy metal ions studied, arsenic, cadmium, and chromium were below detection limit in all the sample sub-areas, however, iron, manganese, and zinc were all detected. Boji-Boji Agbor posted the highest iron value of all, Agbor Obi and Boji-Boji Owa had the least. Manganese is highest in Boji-Boji Owa, and least in Agbor Obi. Again, Boji-Boji Agbor and Alihame recorded the highest and least values of zinc respectively. By implication therefore, and apart from a few rather high metal ion values, there is an obvious description of high heavy metal load in the city centre more than in the suburban remote areas. Indeed this gives credence to the anthropogenic theory of human activities and socio-economic effect having a bearing on groundwater quality.

There are no significant inter-metal relationships from the correlational matrix analysis; hence, not enough information on hydro-geologic heavy metal ions sources and pathways are available for the study area.

Inhabitants of this area are under no immediate or remote health threat from the consumption of this water resource. More so, with the results of the analyses of the chronic daily intake (2 L per day at 72Kg body weight) and the hazard index values pointing very strongly on the wholesomeness of the groundwater relative to heavy metal and arsenic contents, with any short and long-term health implications very unlikely and far-fetched.

It becomes very incisive therefore, from the results of these three heavy metal types (chromium, arsenic, and cadmium) that the people of this study area are rather fortunate. As the *below detection limit* results obtained is a pointer to the wholesomeness of the groundwater aquifer under which they reside. In addition, evidence suggests that levels of arsenic in ground water aquifers in many parts of the world are acceptably below the WHO drinking water guideline (United Nations Environmental Programme Global Environmental Monitoring System and UNEP 2007).

From these results therefore and the parameters studied, it is fitting to conclude and report that the ground water aquifer of Agbor and Owa area generally meet national and international acceptable standards and is therefore adjudged as being safe for drinking with regards to these heavy metals. However, its use for other sundry domestic and agricultural purposes will require further analyses to decipher. We recommend an analysis of individual boreholes to determine any localized areas of poor water quality.
